# A predictive model for stunting among children under the age of three

**DOI:** 10.3389/fped.2024.1441714

**Published:** 2024-09-03

**Authors:** Yuxiang Xiong, Xuhuai Hu, Jindan Cao, Li Shang, Ben Niu

**Affiliations:** ^1^ Department of Medical Information, School of Public Health, Jilin University, Jilin, China; ^2^ Research Development, Shenzhen Health Development Research and Data Management Center, Shenzhen, Guangdong, China; ^3^ Department of Software Technology, School of Software Engineering, Shenzhen Institute of Information Technology, Shenzhen, Guangdong, China; ^4^ Department of Management Science, College of Management, Shenzhen University, Shenzhen, Guangdong, China

**Keywords:** stunting, ages and stages questionnaires, clinical prediction models, nomogram, LASSO

## Abstract

**Background:**

In light of the global effort to eradicate stunting in childhood, the objective of this research endeavor was to assess the prevalence of stunting and associated factors, simultaneously construct and validate a risk prediction model for stunting among children under the age of three in Shenzhen, China.

**Methods:**

Using the stratified random sampling method, we selected 9,581 children under the age of three for research and analysis. The dataset underwent a random allocation into training and validation sets, adhering to a 8:2 split ratio. Within the training set, a combined approach of LASSO regression analysis and binary logistic regression analysis was implemented to identify and select the predictive variables for the model. Subsequently, model construction was conducted in the training set, encompassing model evaluation, visualization, and internal validation procedures. Finally, to assess the model's generalizability, external validation was performed using the validation set.

**Results:**

A total of 684 (7.14%) had phenotypes of stunt. Utilizing a combined approach of LASSO regression and logistic regression, key predictors of stunting among children under three years of age were identified, including sex, age in months, mother's education, father's age, birth order, feeding patterns, delivery mode, average daily parent-child reading time, average time spent in child-parent interactions, and average daily outdoor time. These variables were subsequently employed to develop a comprehensive prediction model for childhood stunting. A nomogram model was constructed based on these factors, demonstrating excellent consistency and accuracy. Calibration curves validated the agreement between the nomogram predictions and actual observations. Furthermore, ROC and DCA analyses indicated the strong predictive performance of the nomograms.

**Conclusions:**

The developed model for forecasting stunt risk, which integrates a spectrum of variables. This analytical framework presents actionable intelligence to medical professionals, laying down a foundational framework and a pivot for the conception and execution of preemptive strategies and therapeutic interventions.

## 1 Introduction

Stunting is conceptualized as a protracted insufficiency of adequate nutritional intake in pediatric populations, thereby impeding linear growth trajectories and culminating in cumulative growth deficiencies (1–3). The initial 1,000 days are a pivotal phase in the early growth and development of children, often resulting in enduring adverse outcomes for those who fail to achieve their full developmental capabilities (4). The prompt identification of developmental disorders holds paramount significance for the welfare of children and their respective families.

The growth and development of children has been investigated and studied in many countries. As per the latest estimates reported in the 2017 Lancet Series, approximately 249 million children (43%) under the age of 5 in low- and middle-income countries (LMICs) faced a heightened risk of suboptimal development in 2010, attributed primarily to stunting or extreme poverty exposure, with the highest concentration observed in South Asia and sub-Saharan Africa (5). A search assessed the nutritional condition of 293 infants and toddlers aged between 0 and 24 months in the Ecuadorian highlands, discovering that stunting was present in 56.2% of the children (6). Furthermore, a comprehensive study revealed that 19.5% of Argentinean children exhibited a predisposition to neurodevelopmental disorders (7). Another study encompassed 330,613 children across 63 nations. Collectively, for all the countries studied, approximately 25% of the children were suspected of having stunt. Regionally, the prevalence spanned from 10% in Europe and Central Asia to a noteworthy 42% in West and Central Africa (8). A prospective birth cohort analysis in Shanghai, China revealed that the overall prevalence of suspected developmental delay at 2, 6, and 12 months of age was 15.6%, 15.8%, and 12.6% respectively (9). An additional investigation disclosed that 14.6% of children exhibited suboptimal scores on the Early Childhood Development Index (ECDI) in the cognitive domain. Furthermore, a positive correlation was observed between these low development scores and the presence of stunting (10). In impoverished regions of China, an alarming 39.7% of children under three years of age are at a heightened risk of developmental delays (11).

To devise effective preventative strategies, it is paramount to gain a comprehensive understanding of the risk factors associated with stunting. Iwayama's research revealed that both advancing maternal and paternal age significantly related to child development and growth in the general populace (12). A comparative analysis of matched groups indicates that being underweight and experiencing a shortened gestation period may contribute to suboptimal weight gain and impaired head growth during infancy (13). Pursuant to the World Health Organization's national assessment of suboptimal infant and young child feeding (IYCF) practices, the duration of breastfeeding is classified as highly satisfactory, whereas the early initiation of breastfeeding and exclusive breastfeeding (EBF) are deemed as satisfactory (14). Animal-sourced foods, which are abundant in essential amino acids, play a crucial role in promoting linear growth and developmental outcomes among young children residing in low- and middle-income countries (15). A research initiative undertaken between 2009 and 2012, encompassing 1,324 children within the age bracket of 0–24 months and domiciled in rural Pakistan, demonstrated that child diet and mother-child interactions improved children's cognitive, language and motor development (16). White matter hyperintensity (WMH) in patients with cerebral small vessel disease (CSVD) is strongly associated with cognitive impairment (17).

The Bayley Scales of Infant Development (BSID) has been a long-standing and extensively utilized instrument for the identification and diagnostic assessment of neurodevelopmental delays among infants. The Bayley-III assessment has attained widespread recognition as the “gold standard” in diagnostic evaluations for early childhood development (ECD) (18). However, BSID is impractical for routine screenings in low-resource settings and must be performed by a trained professional (19–21). The necessity to track the development of large cohorts of children necessitates a reliable yet cost-effective method of assessment. Individual examinations by professionals for each child would be prohibitively expensive. A viable alternative is to directly inquire parents regarding their infant's behavioral patterns. The Parents’ Evaluation of Developmental Status (PEDS), the Child Development Inventories (CDI), and the Ages and Stages Questionnaires (ASQ) have been highly regarded by the American Academy of Pediatrics as outstanding instruments, exhibiting excellent psychometric properties (22). The ASQ is an inexpensive instrument that necessitates an approximate 15-minute time investment for its screening and assessment (23). Furthermore, ASQ has been translated into 16 languages in low- and middle- income countries (LMICs), with at least 23 LMICs having used the questionnaire (24). Many studies have formally addressed the reliability and application of the Ages and Stages Questionnaires 3rd edition (ASQ-3) (25–28). What's more, the United Nations International Children's Emergency Fund (UNICEF) has endorsed the utilization of the ASQ-3, as a means to verify whether children are experiencing normal neurological development (7).

Although there are a number of studies exploring the associated factors of stunting, they all have some limitations. For example, the sample size is mostly concentrated below 2,000, and the data collection area is also relatively concentrated, which may limit their representativeness of the sampling (29, 30). Additionally, few studies have deeply explored the relationship between gestational diseases and developmental delay.

Against this backdrop, the present study comprehensively identified risk factors for stunting, thereby establishing a predictive model with high accuracy. Additionally, it aimed to assess the prevalence of stunting and its associated factors among children under three years of age in Shenzhen, China (31). Nomograms, serving as efficient and precise assessment tools, have the potential to assist clinical medical personnel in objectively identifying children under three years of age who are vulnerable to growth stunting.

## 2 Methods

### 2.1 Sample selection

Participants for the study comprised 9,581 infants and toddlers (0–3 years of age) who received health checkups at the Child Health Clinic of Shenzhen Social Health Center between August 2021 and June 2022. Screening is performed on children and completed by parents or primary caregivers to identify children at risk for developmental delay. The questionnaire investigators were all child health care doctors who were professionally trained and passed the assessment.

### 2.2 Inclusion and exclusion criteria

The following are the criteria for inclusion: (1) infants and toddlers must be between 0 and 36 months; (2) voluntary participation and informed consent; (3) the primary caregiver must be present at the assessment site concurrently with the children. The exclusion criteria include: (1) infants and young children with developmental delays or serious congenital hereditary diseases; (2) parents (caregivers) suffering from intellectual disability or emotional problems; (3) children older than 36 months or refuse to sign the informed consent; and (4) non-primary caregivers present at the assessment site. Based on 9,805 infants under the age of three, 212 individuals with missing main information and 8 individuals with repeated measurements were excluded, and 9,581 individuals meeting the inclusion and exclusion criteria were selected to participate in the analysis.

### 2.3 Outcome variable

The stunt of infants and toddler children were screened and assessed by ASQ-3. ASQ-3 is extensively utilized on a global basis for comprehensive developmental screening of children aged 1–66 months scale (32, 33), and it is the most validated and recommended by the UNICEF to determine whether children have normal neurological development (7). A study has substantiated the high reliability of the ASQ, thereby establishing its efficacy as a screening method for developmental delays (33).

The evaluation results of ASQ-3 are divided into: (1) above the threshold, the total score >2 $\bar{x}$-1 s, indicating that the child is developing normally; (2) near the threshold, the total score is 2 $\bar{x}$−2 s∼2 $\bar{x}$−1 s, indicating that the child may need additional help in one or more areas, but do not show obvious abnormalities and require further monitoring; (3) below the threshold, the total score is <2 $\bar{x}$−2 s, indicating an abnormal development of the child. The ASQ results in one or more areas below the threshold indicate developmental abnormalities, while those above and near the threshold are normal.

### 2.4 Variables

Collect information about infants and their parents through the basic information table, mainly include:

(1) Basic information of children: age and gender;
(2) Birth status: gestational week, mode of delivery, birth weight and birth order;
(3) Feeding situation: feeding mode within six months of age (Feeding methods of infants within 6 months of age), these include exclusive breastfeeding (only the mother's milk without any other dairy or animal milk), mixed feeding (breastfeeding along with milk powder), and bottle-feeding (only milk powder). Complementary food (Food other than breast and milk powder in infancy) and colostrum feeding (Breast milk secreted by the mother within 2–3 days after delivery);
(4) Parental information: parental education level, parental childbearing age, maternal employment status, occupation and maternal health status during pregnancy (gestational diabetes mellitus, hypertension during pregnancy, anemia during pregnancy, bacterial vaginitis, placenta previa, prenatal depression);
(5) Family social and economic status: family type, housing area and district;
(6) Others: average parent-child reading time, average daily time of interaction with other children, and average time of outdoor exercise per day.

### 2.5 Data analysis

To balance the risk of overfitting and underfitting, while ensuring that the model can fully learn the data features during the training stage, the partition ratio of the dataset was selected by 8:2. The dataset was randomly partitioned into training and validation sets, with a 8:2 ratio, to ensure appropriate model generalization. The training set facilitated the selection of salient features and the subsequent development of the predictive model. Conversely, the validation set served to evaluate the performance of the trained model. Categorical variables were presented in terms of frequency (percentage), and the chi-square test was employed to compare variations between distinct groups. To address potential collinearity among candidate variables, LASSO regression models were implemented, enabling the identification of optimal predictor variables. Subsequently, a logistic regression analysis was executed to ascertain the odds ratios and their respective 95% confidence intervals in both univariate and multivariate contexts.

In the current investigation, the discriminative capacity of the model was assessed using the area under the receiver operating characteristic curve (AUROC). Additionally, a calibration curve was employed to quantify the concordance between predicted probabilities and actual observations. To evaluate the clinical validity of the model, decision curve analysis (DCA) was performed. All data were processed utilizing the R software package (version 4.3.1). All statistical tests were conducted with two-tailed significance, and a *P*-value threshold of <0.05 was considered statistically significant.

## 3 Results

### 3.1 Descriptive analysis

**Figure 1:**
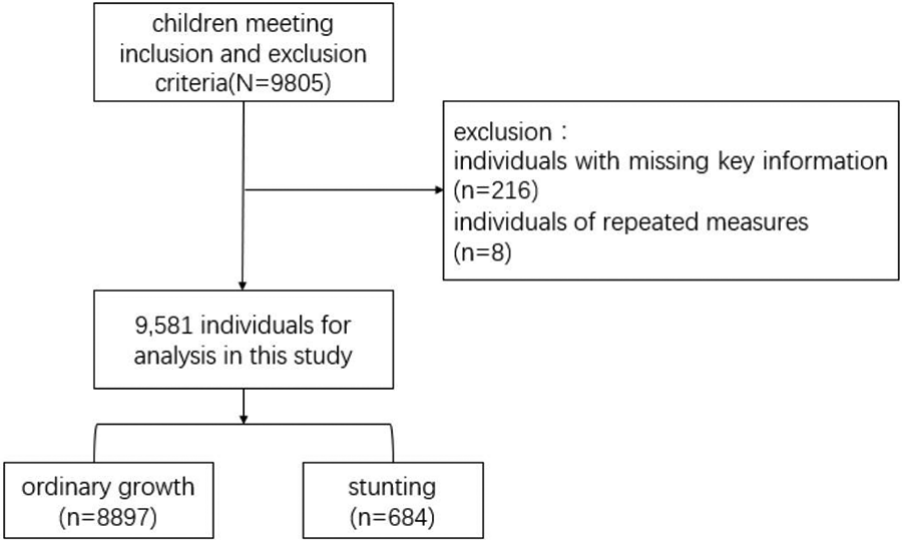
Flowchart of the study.

The cross-sectional analysis included 9,581 respondents, with average age of 13.42 ± 9.16 months (Mean ± SD) and a 43.7% female proportion. The majority of sample’ birth weights were within the normal range (93.2%). Additionally, 49.2% of the infants were exclusively breastfed until 6 months of age. More than half of infants were first-born (55.5%). When considering family factors, it is noteworthy that over half of mothers (56.7%) possess a college education. Concerning social and environmental factors, 11.8% of respondents read more than half an hour per day, while 19.8% spent more than 2 h outdoors per day (Table 1). The selection process of subjects is shown in Figure 1.

**Table 1:** Sample characteristics and prevalence of stunting.

Characteristics	Total (*N *= 9,581)		Normality (*N* = 8,897)	Stunting (*N *= 684)	*p-*value
Gender					<0.01
Male	5,393 (56.3)		4,953 (55.67)	440 (64.3)	
Female	4,188 (43.7)		3,944 (44.33)	244 (35.7)	
Age(months)					<0.01
1–12	5,574 (58.2)		5,221 (58.68)	353 (51.6)	
13–24	2,665 (27.8)		2,474 (27.81)	191 (27.9)	
25–36	1,342 (14.0)		1,202 (13.51)	140 (20.5)	
Birth order	** **		** **		0.006
First-born child	5,315 (55.5)		4,901 (55.09)	414 (59.0)	
Non-first-born child	4,268 (44.5)		1,072 (12.05)	270 (41.0)	
Whether colostrum is consumed					0.011
Yes	1,369 (14.3)		1,249 (14.04)	120 (17.5)	
No	8,212 (85.7)		7,648 (85.96)	564 (82.5)	
Complementary foods is added					0.012
Yes	2,007 (20.9)		1,826 (20.52)	181 (26.5)	
No	7,574 (79.1)		7,071 (79.48%)	503 (73.5)	
Feeding patterns					<0.01
Breast feeding	4,713 (49.2)		4,450 (50.02)	257 (37.6)	
Mixed feeding	3,780 (39.4)		3,456 (38.84)	324 (47.4)	
Bottle-feeding	1,088 (11.4)		985 (11.07)	103 (15.0)	
Health status during pregnancy					0.004
Illness	7,324 (76.4)		6,832 (76.79)	492 (71.9)	
Healthy	2,261 (23.6)		2,069 (23.26)	192 (28.1)	
Mother's education					0.043
Junior high school	2,298 (24.0)		2,110 (23.72)	188 (27.5)	
High school	1,852 (19.3)		1,715 (19.27)	137 (20.0)	
College	5,435 (56.7)		5,076 (57.05)	359 (52.5)	
Father's education					0.083
Junior high school	1,973 (20.6)		1,822 (20.48)	151 (22.1)	
5rr5	2,098 (21.9)		1,931 (21.70)	167 (24.4)	
College	5,510 (57.5)		5,144 (57.82)	366 (53.5)	
Average parent-child reading time per day					<0.01
Less than 5 min	3,196 (33.4)		2,893 (32.52)	303 (44.3)	
5–15 min	3,296 (34.4)		3,084 (34.66)	212 (31.0)	
16–30 min	1,956 (20.4)		1,840 (20.68)	116 (17.0)	
More than 30 min	1,133 (11.8)		1,080 (12.14)	53 (7.7)	
Average interaction time per day with other children	** **		** **	** **	<0.01
Less than 15 min	825 (8.6)		729 (8.19)	96 (11.3)	
15–30 min	1,959 (20.4)		1,790 (2.01)	169 (23.1)	
31–60 min	2,659 (27.8)		2,460 (2.76)	199 (32.7)	
More than 60 min	4,138 (43.2)		3,918 (44.04)	220 (32.9)	
Average outdoor time per day					<0.01
Less than 30 min	1,137 (11.9)		1,019 (11.45)	118 (17.3)	
30–60 min	2,496 (26.1)		2,308 (25.94)	188 (27.5)	
61–90 min	2,352 (24.5)		2,198 (24.70)	154 (22.5)	
91–120 min	1,703 (17.8)		1,587 (17.84)	116 (17.0)	
More than 120 min	1,893 (19.7)		1,785 (20.06)	108 (15.7)	

### 3.2 Prevalence and associated variables of stunting

The prevalence of delayed growth and development was found to be 7.14% in this study. The 18 variables, including the gender, age in months, maternal education, health status during pregnancy, father age, birth order, delivery mode, feeding mode, average parent-child reading time and average outdoor time per day, average interaction time per day with other children, average outdoor time per day, health status during pregnancy, and placenta praevia were significantly different from the normal group (*p* < 0.05) (Table 1).

### 3.3 LASSO logistic regression

**Figure 2:**
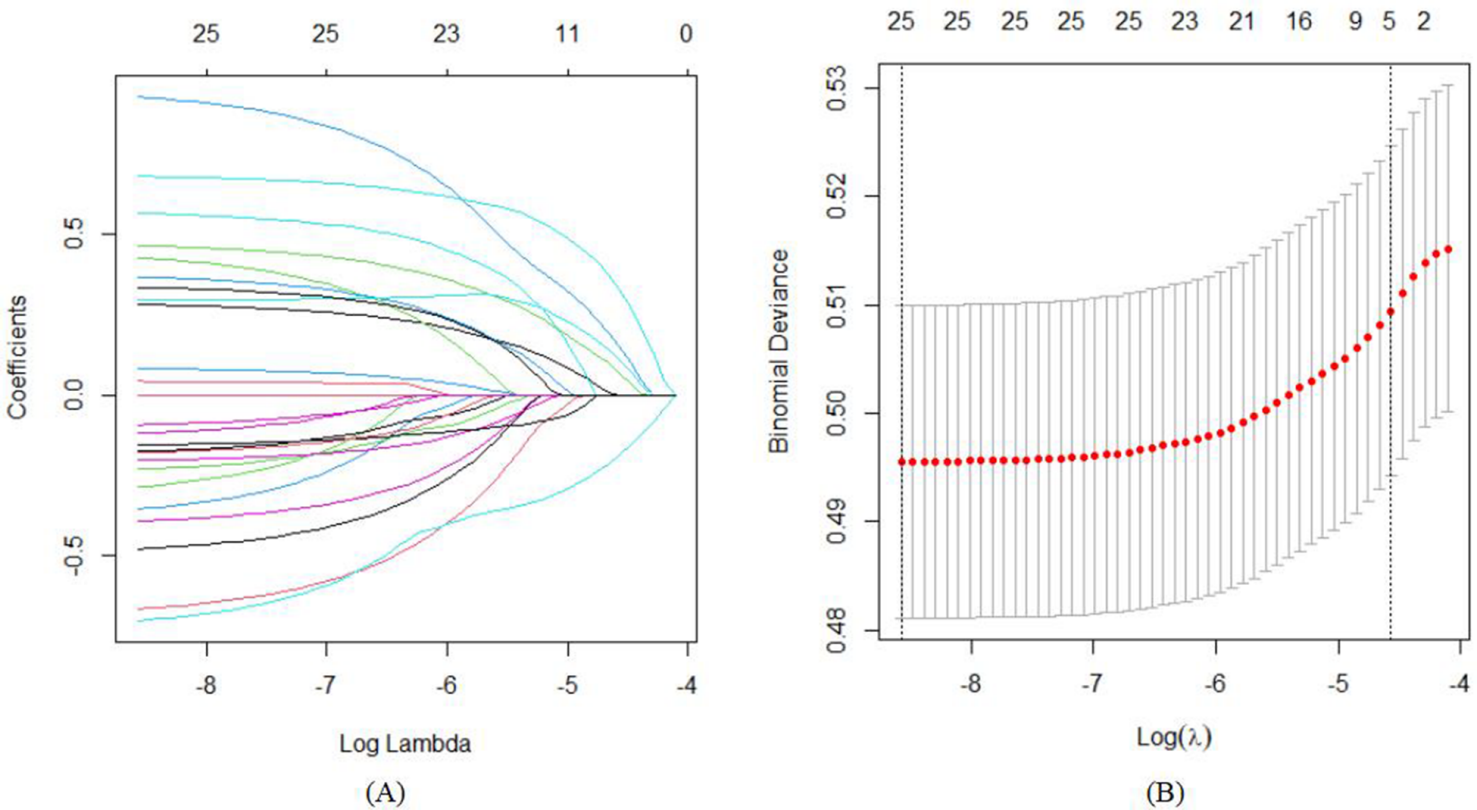
The variable filtering process of the Lasso regression. **(A)** Lasso coefficient profiles of the candidate features; **(B)** the selection of optimal parameters (lambda) by tenfold cross-validation, in which dotted vertical lines were drawn at the optimal values by using the minimum criteria and limits defined by 1 standard deviation.

To reduce noise, variables that proved to be insignificant during the initial univariate analysis were excluded from the LASSO regression. This research utilized a LASSO regression model to pinpoint potential predictors of stunt (Figures 2A,B). Following this, the identified factors linked to stunt were integrated into a logistic regression model. In the end, it was determined that elements like gender, age in months, health status of children, birth order, feeding patterns, average parent-child reading time per day, average interaction time per day with other children and average outdoor time per day were correlated with growth retardation (Table 2).

**Table 2:** Results of binary logistic regression analysis.

Predictive variables	*p-*value	OR (95% CI)
Gender		
Male	Ref	
Female	<0.01	0.75 (0.62, 0.9)
Age (months)		
1–12	Ref	
13–24	<0.01	1.57 (1.23, 1.99)
25–36	<0.01	2.59 (1.99, 3.39)
Health status		
Health	Ref	
Illness	<0.01	1.99 (1.43, 2.76)
Father's age		
<35	Ref	
≥35	0.022	1.34 (1.01, 1.78)
Whether the health is abnormal during pregnancy		
Yes	Ref	
No	0.82	1.2 (0.98, 1.48)
Bacterial vaginitis		
No	Ref	
Yes	0.14	1.6 (0.85, 3.02)
Birth order		
First-born child	Ref	
Non-first-born child	0.07	0.84 (0.7, 1.02)
Delivery method		
Eutocia	Ref	
Cesarean	0.04	0.81 (0.67, 0.99)
Others	0.13	1.43 (0.9, 2.29)
Whether colostrum is consumed		
No	Ref	
Yes	0.87	0.98 (0.75, 1.27)
Whether complementary foods is added		
No	Ref	
Yes	0.30	0.83 (0.59, 1.18)
Feeding patterns		
Breast feeding	Ref	
Mixed feeding	<0.01	1.67 (1.38, 2.04)
Bottle-feeding	<0.01	1.65 (1.22, 2.24)
Average parent-child reading time per day		
Less than 5 min	Ref	
5–15 min	<0.01	0.57 (0.54, 0.83)
16–30 min	<0.01	0.57 (0.46, 0.80)
More than 30 min	<0.01	0.49 (0.35, 0.72)
Average interaction time per day with other children		
Less than 15 min	Ref	
15–30 min	0.043	0.73 (0.54, 0.99)
31–60 min	0.014	0.68 (0.50, 0.93)
More than 60 min	<0.01	0.48 (0.36, 0.65)
Average outdoor time per day		
Less than 30 min	Ref	
30–60 min	0.373	0.88 (0.65, 1.17)
61–90 min	0.247	0.83 (0.61, 1.14)
91–120 min	0.794	1.05 (0.75, 1.46)
More than 120 min	0.178	0.78 (0.55, 1.12)

OR, odds ratio; CI, confidence interval.

### 3.4 Developing predictive models

**Figure 3:**
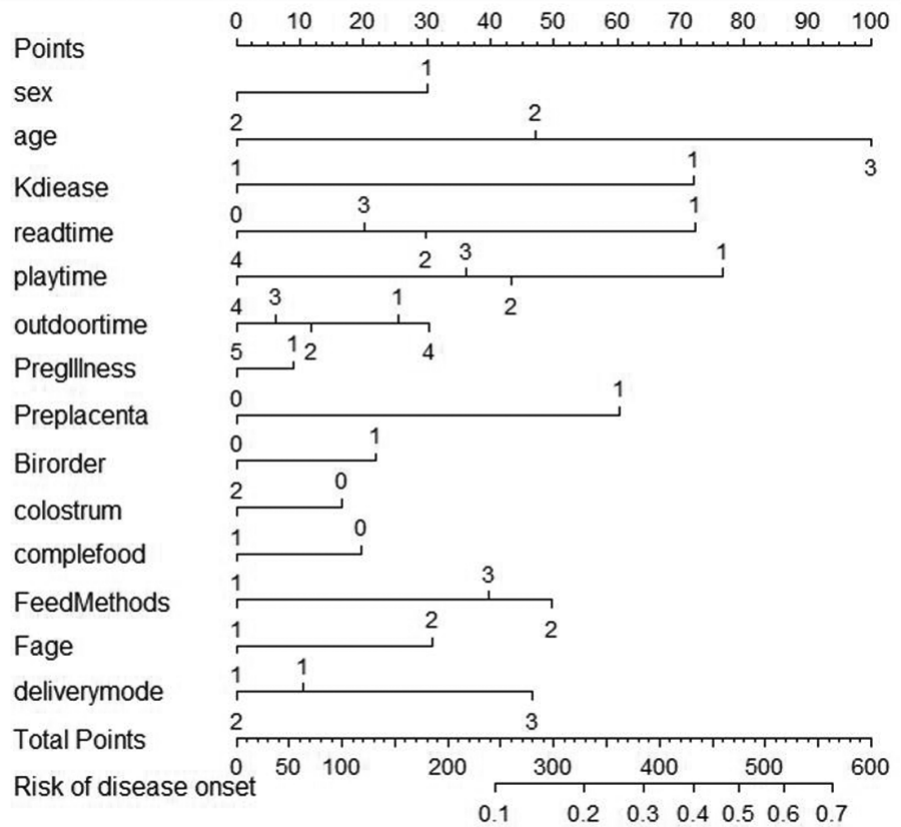
Proposed nomogram for stunt.

By implementing a tenfold cross-validation strategy, the Least Absolute Shrinkage and Selection Operator (LASSO) regression technique was leveraged to discern the most influential predictors that constitute the model's architecture. Subsequently, a comprehensive multiple logistic regression analysis was executed to construct the predictive model. An evaluation of the variance inflation factor (VIF) indicated that all variables possessed VIF values beneath the critical threshold of 4, indicating the absence of multicollinearity and a favorable model fit. The model was developed utilizing RStudio, facilitated by the “rms” package, and a nomogram was subsequently created leveraging the “nomogram” function from the same package (Figure 3; Table 3).

**Table 3:** Nomogram of relevant factors in the assignment method.

Risk factors	Assignment
Sex	Male = 1, Female = 2
Age	1–12 = 1, 13–24 = 2, 25–36 = 3
Kdiease	Health = 1, Illness = 2
Readtime	<5 mins = 1, 5–15 min = 2, 16–30 min = 3, >30 min = 4
Playtime	<15 mins = 1, 15–30 min = 2, 31–60 min = 3, >60 min = 4
Outdoortime	<30 mins = 1, 30–60 min = 2, 61–90 min = 3, 91–120 min = 4, >120 min = 5
Pregillness	Health = 0, Illness = 1
Preplacenta	No = 0, Yes = 1
Birth order	first-born child = 1, non-first-born child = 2
Colostrum	No = 0, Yes = 1
Complementary food	No = 0, Yes = 1
Father's age at childbearing	<35 = 1, ≥35 = 2
Delivery mode	eutocia = 1, cesarean = 2, others = 3

Kdiease: Health status of children; Pregillness: Health status during pregnancy; Preplacenta: placenta praevia; Fage: Father's age at childbearing.

### 3.5 Validating predictive models

#### 3.5.1 Assessment of the model-based discriminative ability

**Figure 4:**
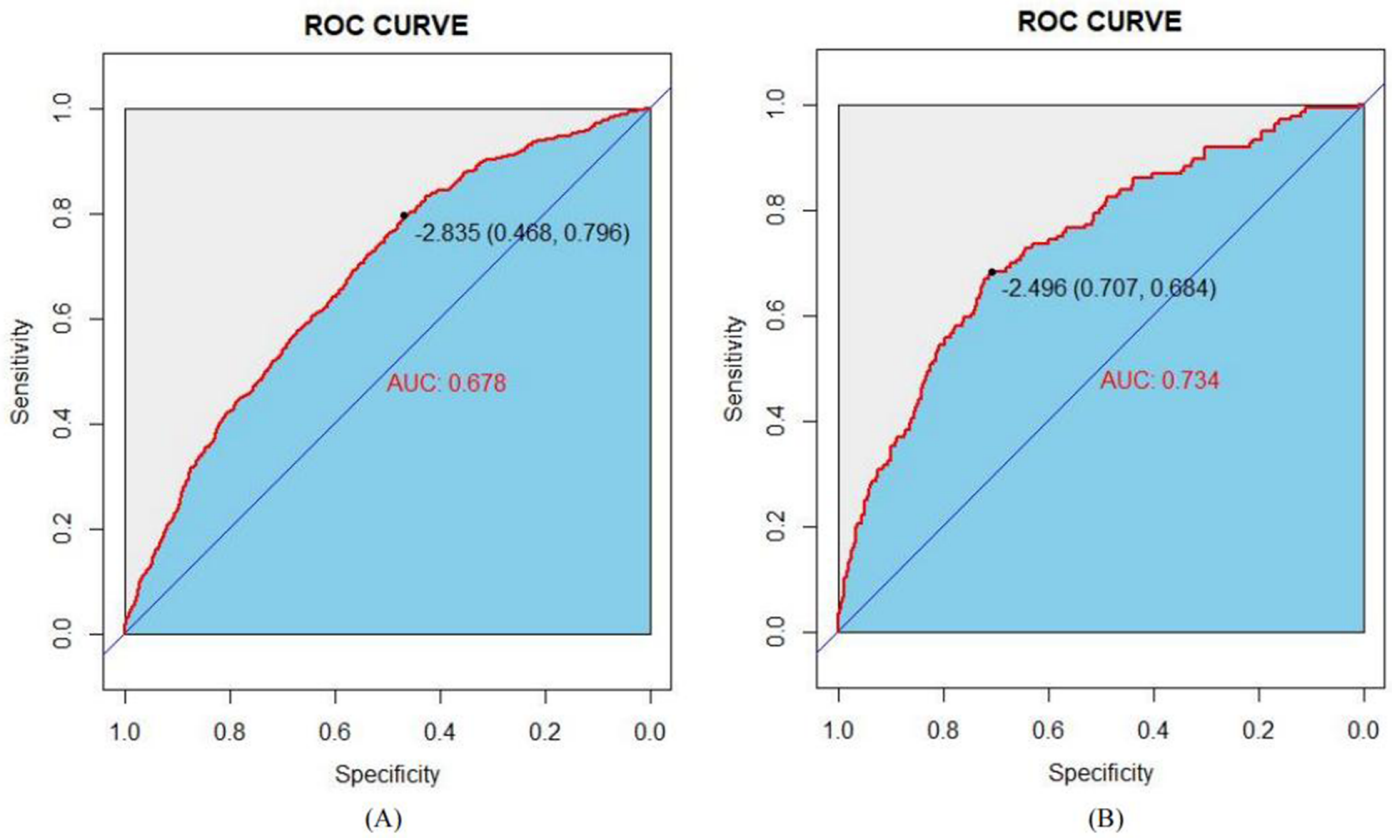
ROC curves of the study's generated nomogram in the study: **(A)** for the training set; and **(B)** for the internal validation set.

The Area Under the Curve (AUC), or area under the ROC curve, serves as a statistical measure to gauge the efficacy of a classification algorithm, highlighting the likelihood that a randomly positive sample will rank higher than a randomly chosen negative sample. This metric is frequently applied to evaluate the performance of machine learning classifiers. In this context, the predictive model's discriminative capacity was appraised by analyzing the occurrence of developmental delays among infants aged 0–3 years in both the training set and the validation set. As illustrated in Figures 4A,B, the AUC for the predictive model, when applied to the training cohort, was calculated to be 0.678 with a 95% confidence interval ranging from 0.6555 to 0.7002. In contrast, the AUC for the validation cohort yielded a slightly higher value of 0.734, accompanied by a 95% confidence interval extending from 0.6892 to 0.7782. The nomogram demonstrates robust discriminative capabilities and predictive accuracy, effectively distinguishing between typical developmental trajectories and growth impediments.

#### 3.5.2 Correcting the predictive model

**Figure 5:**
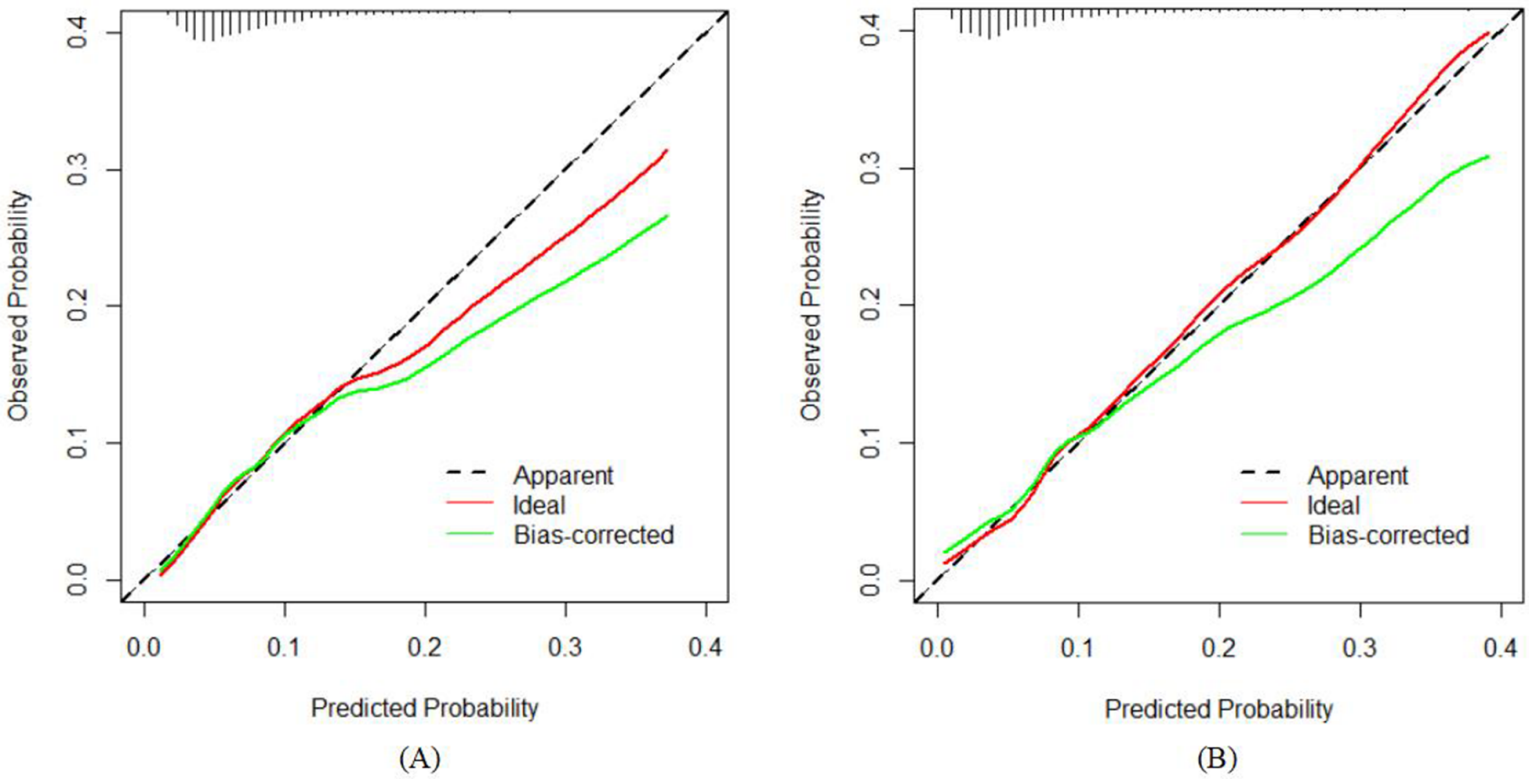
Calibration curves of the nomogram in the study: **(A)** for the training set; and **(B)** for the internal validation set.

The model's congruence was evaluated utilizing calibration plots and the Hosmer–Lemeshow goodness-of-fit statistic, where a non-significant *p*-value (greater than 0.05) indicates a superior fit. The outcomes of the goodness-of-fit test revealed that the model exhibited commendable calibration for the training dataset (*χ*^2^ = 6.2051, df = 8, *p* = 0.6243) as well as for the validation dataset (*χ*^2^ = 11.794, df = 8, *p* = 0.1606). The graphical representations of the calibration curves for both the training and validation cohorts, derived from the logistic regression analysis, are delineated in Figures 5A,B, respectively.

#### 3.5.3 Clinical validity assessment

**Figure 6:**
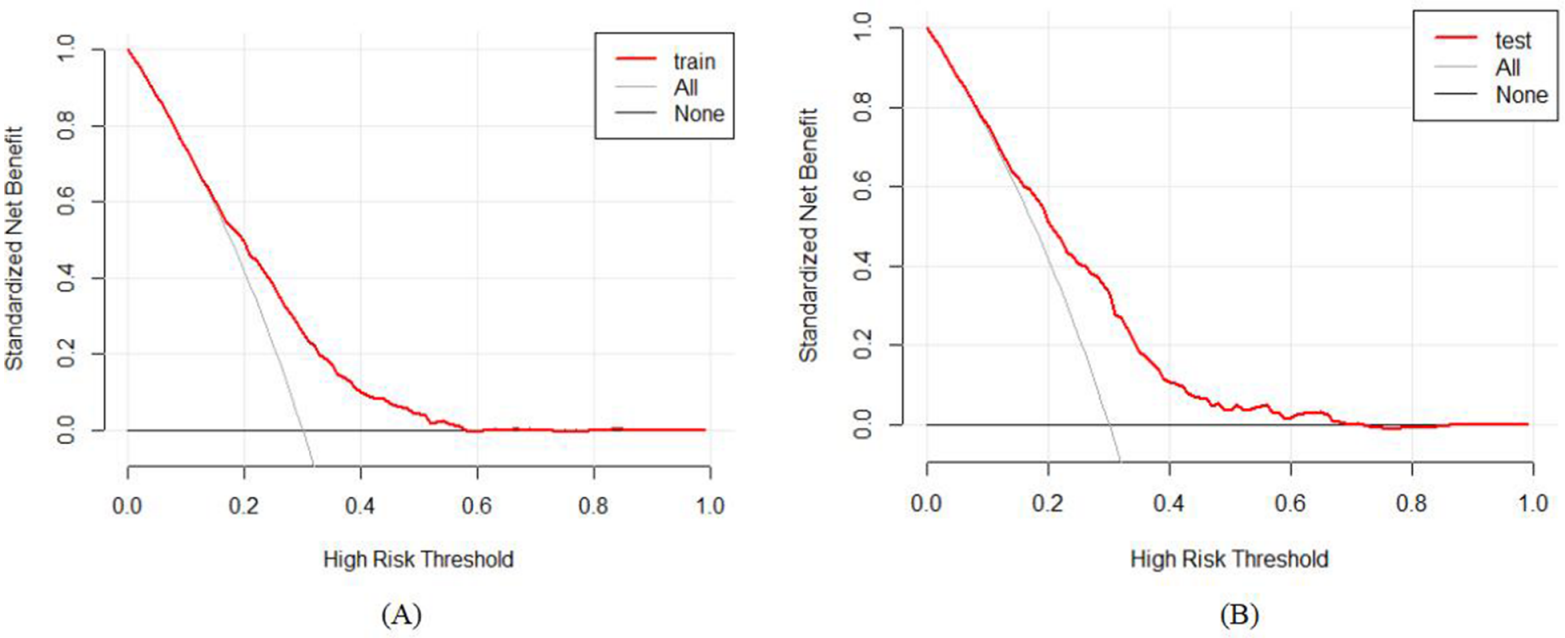
Decision curve analysis (DCA) for the study's nomogram: **(A)** for the training set; and **(B)** for the internal validation set.

The practical applicability of the model was appraised through the Decision Curve Analysis (DCA) framework, with the graphical depictions presented in Figures 6A, B. The DCA demonstrated that the net benefit accruing from the predictive model within the internal validation group markedly surpassed that of the two null hypotheses scenarios. This finding intimates that the nomogram-based model affords a heightened net benefit and precision in predictions, underscoring its clinical significance and practical utility.

## 4 Discussion

This study revealed that the prevalence of stunting among children under the age of three at 7.14% by employing stratified sampling to select 9,581 study participants. In this retrospective study, we developed and valided a nomogram to predict the stunting among children under the age of three. This model has better discrimination ability, clinical applicability and calibration based on AUC, DCA, Calibration plots and the Hosmer–Lemeshow good-ness-of-fit test. Moreover, the logistic regression analysis demonstrated that age, gender, health status, birth order, feeding patterns, average parent-child reading time per day were the best predictors of stunting.

This research demonstrated a correlation between stunting and gender, highlighting a predisposition towards stunt among the male population, thereby corroborating previous findings reported by Hong Zhou and colleagues (34). The rationale behind the conducted analysis potentially stems from the speculation that stunting is associated with genetic inheritance or mutational alterations within genes. Furthermore, our study revealed no significant association between stunt and factors such as birth weight, maternal gestational diabetes, or gestational hypertension. However, contrasting research has indicated that low birth weight, maternal gestational hypertension, premature rupture of membranes (PROM), and smoking during pregnancy are all contributory factors that elevate the risk of neurodevelopmental impairments (NIs). Conversely, EBF and a higher socioeconomic status appear to be protective factors that mitigate this risk (35). New evidence has emerged, suggesting that antenatal depression, or maternal depression experienced during pregnancy, holds profound implications not only for the mother's own well-being but also for the emotional and behavioral trajectory of the developing child. This finding underscores the intricate link between maternal mental health and the future psychosocial development of offspring (36).

Our research has illuminated the significance of feeding patterns as predictors of stunting, with numerous studies corroborating the unequivocal benefits of EBF on child development. A cluster-randomized controlled trial, conducted in the western Kenyan sub-county of Bondo, revealed noteworthy positive correlations between EBF during the 3- to 6-month age window and various aspects of child development, particularly in the domains of communication, gross motor skills, and problem-solving abilities (37). In a study conducted by Wallenborn JT and his team, it was observed that adhering to the World Health Organization's (WHO) recommendations for EBF remains crucial for promoting healthy physical growth and cognitive development, even in environments where complementary foods are readily accessible (38). A comprehensive cohort analysis conducted in rural South Africa has revealed that EBF is associated with a reduced prevalence of conduct disorders and a modest correlation with enhanced cognitive development among male children (39). A prospective birth cohort study originating from Brazil has unveiled that breastfeeding is positively correlated with superior performance in intelligence assessments administered three decades later, potentially having a significant impact on real-life outcomes, including the augmentation of educational attainment and income during adulthood (40). Furthermore, a Polish Mother and Child Cohort Study failed to identify any significant association between the duration of breastfeeding and child development (41). A study conducted among Chinese toddlers aged 1–3 years revealed a negative correlation between feeding difficulty and their overall health and development (42). For this reason, breastfeeding during infancy is highly emphasized as a pivotal strategy to foster growth and development, even among healthy young children (43).

Concurrently, this study also noted a weak association between the mode of delivery and the incidence of stunting. Prior research has identified certain adverse outcomes associated with cesarean delivery. Specifically, it has been linked to a heightened risk of Attention Deficit Hyperactivity Disorder (ADHD) and Autism Spectrum Disorder (ASD) (44, 45). Despite this, the current research evidence remains inconclusive in determining the precise effect of cesarean section on child health outcomes. For example, certain studies have suggested that cesarean delivery does not markedly increase or decrease the likelihood of suspected developmental delay in children compared to vaginal delivery (46–48). In addition, this study showed that maternal educational level is also associated with stunt. A study conducted in Uganda revealed that children whose mothers possessed secondary education exhibited lower odds of stunting and underweight compared to children whose mothers had no formal education (49). A comparative cross-sectional study has revealed that the prevalence of stunting and wasting is comparatively lower among children of employed mothers than among those of unemployed women (50). In view of this, the reasons for this difference require more intensive research.

Finally, our study also found that reading time and interacting time with others children was significantly asscociated with the occurrence of stunt, which is consitent with previous research findings. A comprehensive study has revealed a significant positive association between augmented reading time across multiple time intervals and enhanced ASQ-3 scores, encompassing fine motor, gross motor, personal-social, and comprehensive development domains, over a protracted period (26). A prospective longitudinal cohort study in Canada has uncovered that toddlers’ exposure to informal play opportunities, reading picture books, and supervision in childcare centers emerges as protective factors mitigating the risk of delayed speech development (51). The results from the China ECD Program have additionally demonstrated that a deficiency in books and toys (52), coupled with inadequate learning activities (53), substantially elevate the risk of developmental delays in children aged 0–35 months residing in rural areas. Furthermore, a longitudinal birth cohort study has revealed that responsive caregiving and learning opportunities serve as protective factors for young children, mitigating the negative impacts of early adversities on their adolescent human capital development (54).

Constructed through regression analysis, the nomogram integrates diverse predictive indicators to visually represent the inter-variable relationships within the predictive model. The visualization is accomplished via the placement of scaled line segments on a unified plane, adhering strictly to a predetermined ratio. This serves as a predictive instrument that forecasts the likelihood of a specific clinical outcome by aggregating the scores attributed to individual predictors, thereby yielding an overall score. The development of a prediction model for stunting among children under three years of age represents a novel contribution of this study. Notably, the nomogram quantitatively translates hazard ratios into scores, facilitating the straightforward calculation of outcomes. This approach enables an individualized risk assessment for each individual, thereby enhancing both relevance and accuracy.

This study had several limitations. Firstly, the employment of a cross-sectional design in this study limited the capacity to establish definitive causal relationships. Secondly, the nomogram devised in this study is tailored specifically to Chinese data, and its applicability to other regions and countries necessitates further determination through external validation. The data originates from the verbal reports of the infants’ parents; however, the parents may not be fully aware of their child's actual condition, which could result in discrepancies between the data and the actual situation.

This study had some strengths. Firstly, this study has a large and representative sample size. Secondly, we explored the relationship between gestational diseases and developmental delay.

## 5 Conclusion

Our stunt risk prediction model provides a reliable and accurate tool for children under the age of three in Shenzhen, China. This model serves as a valuable asset to clinical practitioners by furnishing a theoretical foundation and a point of departure for the development of preemptive prevention and intervention strategies.

## Data Availability

The original contributions presented in the study are included in the article/Supplementary Material, further inquiries can be directed to the corresponding author.
